# Impact of an 18F-FDG PET/CT Radiotracer Injection Infiltration on Patient Management—A Case Report

**DOI:** 10.3389/fmed.2018.00143

**Published:** 2018-05-15

**Authors:** Jackson W. Kiser, James R. Crowley, David A. Wyatt, Ronald K. Lattanze

**Affiliations:** ^1^Carilion Clinic, Roanoke, VA, United States; ^2^Lucerno Dynamics, Cary, NC, United States

**Keywords:** PET-CT, extravasation of diagnostic and therapeutic materials, SUV, FDG, time activity curve

## Abstract

Major management decisions in patients with solid tumors and lymphomas are often based on 18F-fluorodeoxyglucose (18F-FDG) PET/CT. The misadministration of 18F-FDG outside the systemic circulation can have an adverse impact on this test's sensitivity (1) and is not uncommon (2–7). This report describes how an 18F-FDG misadministration led to a repeat PET/CT study, resulting in the visualization of distant metastases that changed the original treatment plan. The findings suggest that routine injection monitoring is indicated whenever sensitivity is critical, and support claims that infiltrations can confound interpretation of semi-quantitative PET outcome measures in patients who are followed longitudinally (2).

Precision medicine has been increasingly in the news^1^. A year-by-year review of The New England Journal of Medicine shows the term “precision medicine” has increased over 15-fold from 2012–2013 to 2016. Positron Emission Tomography (PET), a crucial imaging tool in oncology care (8), plays a vital role in precision medicine. In 2017, it had been projected that nearly 3 million PET/CT scans would be performed in the US, with ~2.9 million scans used for oncology applications (9). While coordinated efforts to develop and encourage the adoption of guidelines and standards continue to minimize variability in molecular imaging results (10, 11), the radiotracer injection process remains susceptible to error.

The seminal quantitative 18F-FDG PET studies in the 1970's and 1980's were based on bolus injections (12). This practice continues today with an emphasis on precision. Guidelines continue to prescribe quality assurance procedures that have relatively small influence on variability such as: measuring residual activity after injection and synchronizing clocks (11). However, errors in the physical delivery of the 18F-FDG have the potential to introduce significant variability (1). These para-venous injections are known as infiltrations or extravasations. While only a few centers have published or presented on infiltrations, a critical review shows their aggregated rate is about 15% (423 infiltrations in 2,802 patients (2–7).

In an 18F-FDG administration that conforms to expectations, the entire injected dose is delivered to systemic circulation, and therefore, is the same dose used in the Standardized Uptake Value (SUV) calculation. An infiltration creates an unknown mismatch between the calculated injected dose and the actual dose delivered in circulation. Some of the infiltrated dose remains near the injection site and returns to systemic circulation largely through lymphatic reabsorption at an unknown rate during the prescribed uptake period. These problems alter the supply and clearance of the radiotracer to the tissue in an unknown way and reduces the calculated SUV. The quality of the image can also be degraded by an infiltration through the delivery of an inadequate 18F-FDG dose and through the continuous recovery of the radiotracer from the infiltration site, without enough time for subsequent clearance in the circulation.

Appropriately characterizing the quality of the radiotracer injection is difficult. While abnormal uptake at the injection site is evidence of an infiltration (11), the PET/CT field of view (FOV) often excludes the injection site (2). When an injection site is in the FOV, visualization and measurement of abnormal uptake may underestimate the true extent of an infiltration, since PET/CT static images are acquired ~60 min post-injection and cannot represent the state of infiltration as it resolved during the uptake period (13). A recent presentation, demonstrating dynamic PET/CT images and time-activity curves (TACs) from the uptake period, highlighted “invisible infiltrations” and how their visual evidence resolves completely before the acquisition of static PET/CT images (14). These invisible infiltrations appear as prolonged venous stasis and also contribute to the difficulty in characterizing 18F-FDG administrations using the PET/CT image. These issues suggest that the field needs an effective means to monitor the quality of the administration.

Our nuclear imaging facility is participating in a multi-center quality improvement project that uses a novel device to characterize each 18F-FDG injection. The goal is to reduce the overall infiltration rate. The device provides real-time quality control and addresses these issues that tend to confound proper injection assessment by providing TACs from the injection site during the uptake period. After a period of extended use, the device's quality assurance software provides an analysis of injection techniques and factors that can help improve a facility's performance. We encountered this case during this project.

## Case presentation

**Figure 1:**
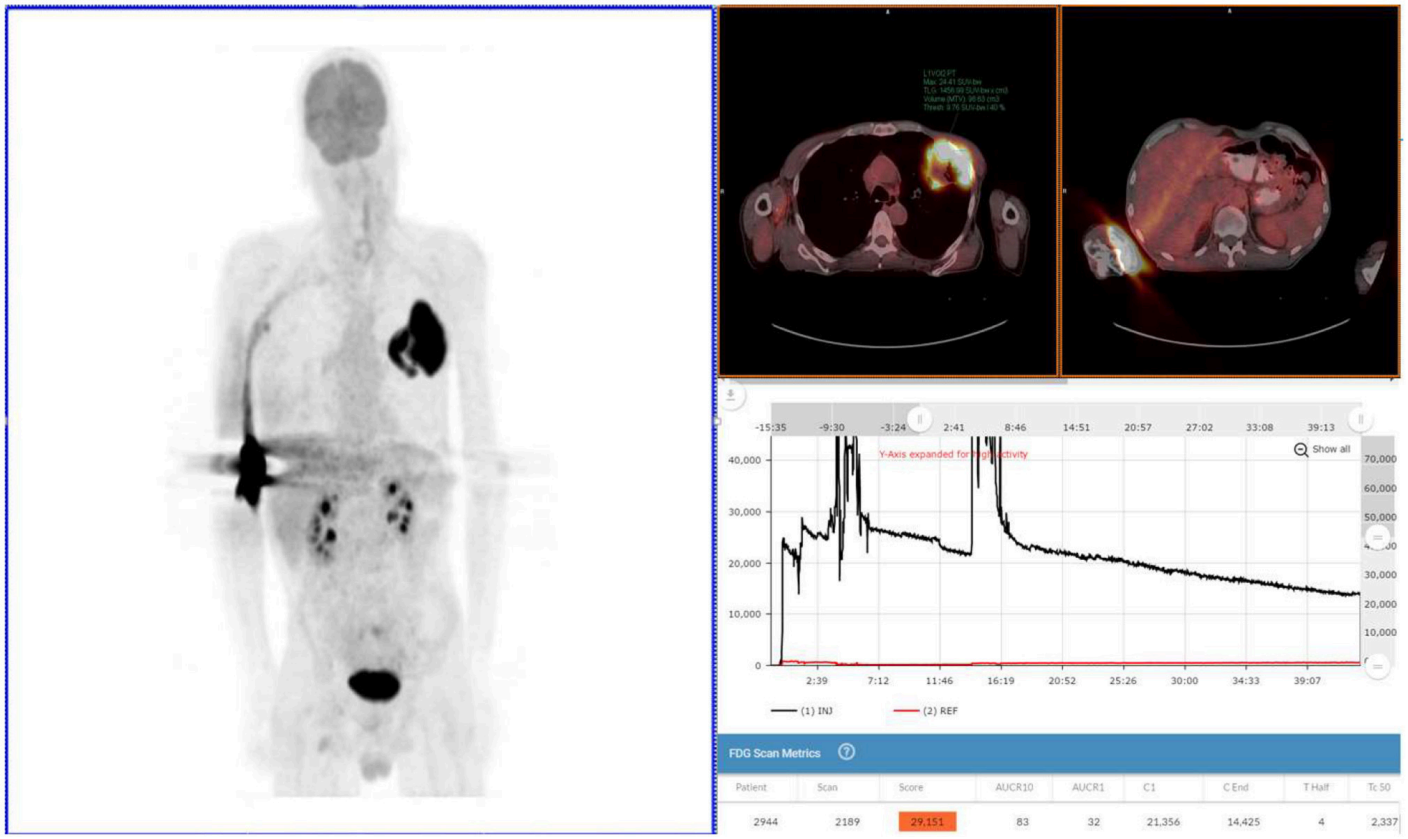
Exam 1—maximum intensity projection image, fused axial images, and time-activity curves of baseline PET/CT scan. MIP image (above left image), taken 61 min post-injection, reveals lung mass, and abnormal uptake at right antecubital fossa (radiotracer injection site) and right axilla uptake thought to be the result of the infiltration. Black time-activity curve (lower right image) from injection arm sensor reveals severe infiltration during the first 40 min of the uptake period. Red time-activity curve (also lower right image) from contralateral arm sensor reveals minimal uptake. Fused PET/CT images (upper right images) reveal left upper lobe mass (SUV_max_ 24) and infiltration artifacts at adrenal level. No evidence of metastatic disease.

In 2017, a 60–70 year-old male who never smoked tobacco presented with chest pain and weight loss, which prompted a chest X-ray and subsequent CT. The CT revealed a left upper lobe mass with no evidence of nodal involvement or metastases. A staging 18F-FDG PET/CT scan confirmed the presence of a single, large, lung mass; however, the quality and quantification of the image was potentially impacted by a relatively severe infiltration in the right antecubital fossa (Figure 1). The patient was immediately repositioned on the imaging table with their arms over their head for another PET imaging session. This image confirmed the presence of the single, large, lung mass. Biopsy of the mass revealed a non-small cell lung cancer.

**Figure 2:**
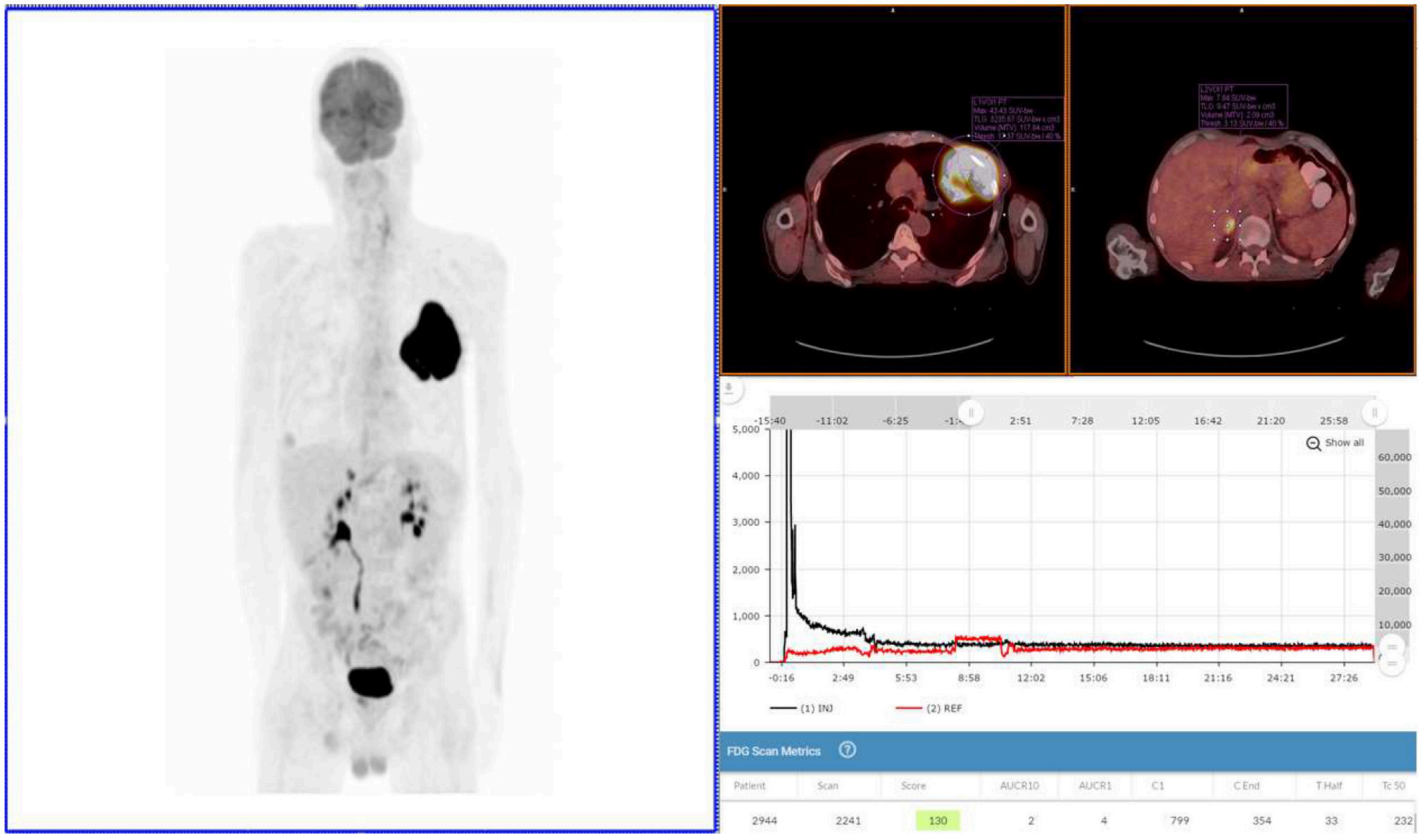
Exam 2—maximum intensity projection image, fused axial images, and time-activity curves of repeat PET/CT Scan. Repeat PET/CT MIP image (above left image), taken 3 days after first PET/CT and 65 min post-injection, reveals lung mass, right adrenal lesion, prostate lesion, and some minimal abnormal uptake in left forearm (radiotracer injection site was left hand). Black time-activity curve (lower right image) from injection arm sensor reveals a nearly ideal injection. Red time-activity curve (also lower right image) from contralateral arm sensor reveals expected uptake. Fused PET/CT images (upper right images) reveal left lobe mass (SUV_max_ 43) and adrenal lesion.

The patient was invited back for a repeat scan 3 days later to confirm initial staging of T3N0M0. The repeat scan (Figure 2) revealed more information about the single lung lesion and also showed new disease: a right adrenal lesion and also a potential prostate lesion, not seen in Exam 1. Based on the new information from the repeated PET/CT scan, staging was revised to T3N0M1.

## Discussion

### Exam 1

The patient received the standard of care pre-PET/CT scan instructions, fasted appropriately and presented with blood glucose levels of 4.7 mmol/L (85 mg/dL). A Certified Nuclear Medicine Technologist (CNMT) gained access in the right antecubital fossa using a 24-gauge needle and IV cannula. A sensor, comprised of a single scintillating crystal paired with silicon photomultiplier, (Lucerno Dynamics, Cary, NC) was placed topically ~7 cm proximal to the access site and a second sensor was placed on the opposite arm in a mirrored location. The patient was manually injected with 635 MBq (~17 mCi) of 18F-FDG and the IV was flushed with 14 mL of saline. After ~40 min, the sensors were removed and the injection data were downloaded. The resulting injection arm TAC revealed a severe infiltration. At the 61 min post-injection mark, emission images were acquired (Exam 1) on a PET/CT scanner (Siemens Biograph 40, Knoxville, TN) with 3D acquisition. A 3-min per bed acquisition time was performed in a cranial to caudal direction. After review of the PET images, the patient was positioned back on the imaging table with arms over their head. At the 92 min post-injection mark, emission images were acquired.

The patient was invited back for a repeat scan 3 days later in accordance with our center's standard practice to repeat scans that may have been moderately or severely infiltrated.

### Exam 2

After fasting appropriately, the patient presented with blood glucose levels of 4.8 mmol/L (87 mg/dL). A CNMT, different from the one 3 days earlier, gained access in the left hand using a 24-gauge needle and IV cannula. Sensors were placed ~7 cm proximal to the access site and on the opposite arm in a mirrored location. The patient was then manually injected with 572 MBq (~15.5 mCi) of 18F-FDG. The IV was flushed with 20 mL of saline. After ~30 min the sensors were removed from the patient and the injection data downloaded. The resulting injection arm TAC revealed a satisfactory 18F-FDG injection. At the 65 min post-injection mark, Exam 2 was performed with identical acquisition, processing parameters, and image analysis software using the same PET/CT scanner.

## Results

Exam 1 revealed abnormal uptake at the right antecubital fossa, indicative of a severe infiltration, and a lung lesion with a SUV_max_ of 24. Right axilla uptake was noted, but suspected to be associated with infiltration. No other lesions were noted in the 61 min post-injection images nor in the 92 min post-injection images with arms over the head. Based on biopsy results and the image review, the patient was diagnosed with squamous cell lung cancer and staged as T3N0M0. Surgical debulking and adjuvant therapy were considered as options after discussion with a cardiothoracic surgeon.

Exam 2 revealed the lung lesion with a SUV_max_ of 43, an 80% increase from the Exam 1. A right adrenal lesion was highly suspicious with a SUV_max_ of 11. Another lesion was detected in the prostate region with a SUV_max_ of 5. The significance of the prostate lesion was uncertain in the setting of lung cancer and not strongly considered to represent metastatic disease in this case. No activity was noted in the right axilla, confirming the impact of the infiltration in Exam 1. Based on the image review, staging was altered to T3N0M1, and the planned patient management was changed. A comparison of Exam 1 and Exam 2 can be found in Table 1.

**Table 1:** Comparison of exam 1 and exam 2.

**18F-FDG PET/CT Exam 1**	**18F-FDG PET/CT Exam 2 (~73 h later)**
Blood sugar: 4.7 mmol/L (85 mg/dL)	Blood sugar: 4.8 mmol/L (87 mg/dL)
Injection to scan time: 61 min	Injection to scan time: 65 min
Lung lesion SUV_max_: 24	Lung lesion SUV_max_: 43
Adrenal: not initially observed and indeterminate in retrospect	Adrenal: SUV_max_: 11
Staging: T3N0M0	Staging: T3N0M1
Surgical candidate: Possibly	Surgical candidate: No

*SUV, Standardized uptake value*.

## Concluding remarks

18F-FDG PET/CT has played an increasingly important role in the initial staging of cancer and the assessment of tumor response (15). In precision medicine, this tool will play an even more significant role, using semi-quantitative, or quantitative PET/CT data for single or multi-time-point assessments (e.g., diagnosis, staging, eligibility assessment, or investigation of predicative or prognostic biomarkers) (10).

An infiltration will negatively affect image quality and underestimate an SUV. Depending on the purpose of the scan and awareness of an infiltration, a clinician may be able to salvage some infiltrated scans by delaying imaging 120–180 min post-injection. The extended uptake period may yield better images. However, since an infiltration will confound quantification measures based on the severity of the infiltration, repeating the scan may be a better option. Since multiple PET/CT scans—each requiring an 18F-FDG injection—are used to assess response, the cumulative probability an infiltration will impact the assessment increases with the number of scans. In the current nuclear medicine practice, injections sites that are out of the FOV, invisible infiltrations, and visible infiltrations underestimated due to the static nature of images, can all contribute to the interpreting and treating physicians reaching the wrong conclusion about staging and tumor response to treatment. This single case report demonstrates the major impact an 18F-FDG infiltration can have on PET/CT SUV values and on patient staging; it also suggests that routine injection monitoring is indicated whenever sensitivity of PET/CT scanning is critical.

## Ethics statement

Extensive efforts were made by the authors to contact the next of kin for consent to the publication of this case report, however this could not be obtained. Efforts have therefore been made to ensure that the case is presented with the minimal amount of potentially identifiable information.

## Author contributions

JK: provided images, image analysis, and helped draft the initial submission; JC: provided details of the patient imaging sessions and provided the initial draft of the submission; DW: provided patient details and staging information, and staging considerations; RL: provided time activity curves, created subsequent drafts of the submissions, and coordinated the editing process.

### Conflict of interest statement

RL was employed by company Lucerno Dynamics. The other authors declare that the research was conducted in the absence of any commercial or financial relationships that could be construed as a potential conflict of interest.

## Footnotes

^1^Google Trends Search. Available online at https://trends.google.com/trends/explore?date=all&q=precision%20medicine (Accessed August 27, 2017).
